# The Impact of a Postgraduate Learning Experience on the Confidence of General Dental Practitioners

**DOI:** 10.3390/dj5020016

**Published:** 2017-04-26

**Authors:** Peter Fine, Chris Louca, Albert Leung

**Affiliations:** 1 UCL Eastman Dental Institute, Department of CPD, London WC1X 8WD, UK; albert.leung@ucl.ac.uk; 2 University of Portsmouth Dental Academy, Portsmouth PO1 2QG, UK; chris.louca@port.ac.uk

**Keywords:** confidence, postgraduate, educational experience, general dental practitioners

## Abstract

This study aimed to explore the relationship between participating in a learning experience and the ensuing changes in confidence. A self-selected group of General Dental Practitioners (GDPs) entered a five-year, part-time postgraduate master’s training programme in restorative dentistry. Confidence in communication with patients and technical skills were measured at the start of the programme by questionnaire and at the conclusion of the programme by questionnaire and personal interview. A total of 72 clinicians started the programme; 27% (*n* = 20) completed the master’s degree. Assessment of confidence revealed a spread from 4/10 to 10/10 for communication with patients and clinical skills in restorative dentistry before the programme started. A total of 15% (*n* = 11) volunteered for interview. Analysis of qualitative data revealed (i) a perceived increase in confidence from all clinicians; (ii) a perceived greater ability to treat patients; (iii) an increase in treatment options being offered to patients; (iv) a perceived increase in treatment uptake by patients; and (v) greater job opportunities. The study showed a positive relationship between the learning experience and the perceived increase in confidence of clinicians. The increase in confidence manifested itself in better communication and clinical skills.

## 1. Introduction

The aim of this study was to look at the effect of a five-year learning experience on the perceived confidence of a group of clinical professionals. The study used a self-selected group of General Dental Practitioners (GDPs) who completed a five-year, part-time postgraduate programme leading to a master’s degree.

The study investigated the impact of a change in confidence on this self-selected cohort and considered the link between their perceived confidence and an educational intervention or learning experience gained through participation on a part-time master’s degree in restorative dentistry. The programme adopted traditional teaching methods; it was flexible and was designed for working clinicians, with multiple exit points. It awarded qualifications ranging from a Postgraduate (PG) Certificate after one year of study, to a PG Diploma after a further two years and culminated in a master’s degree after another two years. The course content focused on the repair and replacement of damaged and/or missing teeth.

Clinical Professionals have been reported to carry out their professional tasks more comfortably and effectively when they perceive themselves to be confident [1] (pp. 9–28). Familiarity with a task usually increases levels of confidence [2]. However, various factors, such as changes in context or the task itself could have an adverse impact on individuals’ self-confidence [2] (e60–e65).

The Oxford English Dictionary defines confidence as ‘a feeling of self-assurance arising from an appreciation of one’s own abilities or qualities’ [3]. Bandura (1986) substituted the term self-efficacy for confidence and described self-efficacy as ‘peoples’ judgements of their capabilities to organise and execute courses of action required to attain designated types of performance’ [4] (p. 19). Self-efficacy can be seen as the confidence that people have in their ability to achieve what they set out to do [5]. Pajares (1997, 2002) applied scales of self-efficacy to educational research, primarily in studies of academic motivation and self-regulation [6,7]. These scales of self-efficacy are important in measuring the strength and generality of self-efficacy, which have an impact on the confidence of individuals in this study. The more self-efficacious they are, the more likely they appear to progress through the programme. The link between self-efficacy and confidence was summarised by Crozier (1997): “self-efficacy research has helped to tease out the contributions that ability and self-confidence in one’s ability make to academic success and in careers beyond education” [8] (p. 167).

The concept of self-confidence is under-pinned by two rationales: (i) an assessment of one’s knowledge and skills of a situation or task, based on previous experience and (ii) a review of the situation using a level of belief about how successful one will be [9] (pp. 107–118).

The aim of this study was to investigate the changes in perceived confidence levels amongst a cohort of GDPs in relation to their ability to perform dental restorative tasks and to communicate with their patients.

## 2. Materials and Methods

Ethics approval was not required having sought the advice of the relevant university committee. A mixed-method approach to data collection was employed. Quantitative data were collected throughout the duration of a five-year, part-time master’s programme—at the conclusion of the certificate, diploma and master’s stages of the programme—by a series of linked questionnaires with up to 24 questions, sharing some common themes. The questionnaires used a combination of Likert scale statements and structured/semi structured/multiple choice questions. The use of pilot questionnaires with participant feedback, informed the final versions of the questionnaires used to investigate the perceived confidence of the participants. Hard copies of all the questionnaires were delivered to the course participants by administrative staff and on completion, were collected in person by the same staff. The participants were advised about the nature of the study and gave their full consent. The quantitative data were entered into a spreadsheet and analysed using SPSS (Version 21, IBM SPSS Software 2015).

A pre-course questionnaire was used to establish confidence levels prior to attending the programme. Three further questionnaires were used at the completion of the certificate, diploma and master’s elements of the overall programme, in order to record progressive changes in confidence. All questionnaires enquired about (i) demographic information; (ii) the perceived impact of the programme on learning experience; (iii) the perceived impact of changes in confidence on clinical practice; (iv) plans for future learning and (v) the preferred teaching methods employed. The various questionnaires used shared common questions in order to investigate changes in perceived confidence on progression through the programme, as well as more bespoke questions relevant to the different stages of the programme.

Qualitative data were additionally collected via the (i) same questionnaires; (ii) focus group discussions; (iii) personal interviews and (iv) field notes. Each focus group discussion was transcribed as a general report and personal interviews were transcribed verbatim, by one of the authors (PF).

The qualitative data were organised using a ‘Framework’ spreadsheet, which allowed for the transcribed interviews, focus group discussions, field notes and comments on the questionnaires, to be arranged under dominant themes that had emerged during transcription [10]. Pre-determined semi-structured questions were used during the interviews and focus group meetings. Relevant accessory questions were used where appropriate. The questions for the interviews were designed following analysis of the results from all the questionnaires.

Qualitative data were analysed using an approach which investigated the study participants’ perceptions, perspectives and interpretations of a particular situation (or phenomenon), referred to as a phenomenological analytical approach [11] (pp. 22–250). A thematic analysis was undertaken following organisation of the data into a ‘Framework’ [10] spreadsheet. The ‘Framework’ system of organising qualitative data allowed for a more logical, thorough analysis of large quantities of data.

## 3. Results

Both quantitative and qualitative results are represented here.

### 3.1. Quantitative Results

The number of returned and completed questionnaires were as follows: (i) Pre-course 72/72 (ii) Certificate 50/72; (iii) Diploma 22/30 and (iv) Master’s 18/20. The final questionnaire was distributed to the 20 participants who completed the full master’s programme; of which 18 were returned and these questionnaires contributed to the final analysis. The demographic results are described in Table 1.

Table 1 illustrates the diversity of the sample of GDPs in the study. This sample size is considered to be appropriate for this type of study, as indicated by Cresswell (1997) [12]. It was observed that the practice profile of the participants changed from an initial state sponsored National Health Service approach to a more independent style of practice.

Table 2 shows the results from the pre-programme questionnaire indicating why the participants decided to embark on the master’s programme. Increased confidence along with increasing knowledge and skills were the main reasons for attendance.

**Figure 1:**
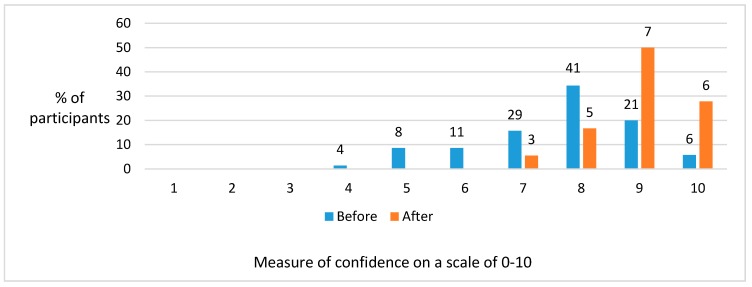
Changes in confidence levels (where 0 = no confidence and 10 = total confidence) with respect to communication skills before and after the programme. The numbers above the columns are actual numbers of participants.

**Figure 2:**
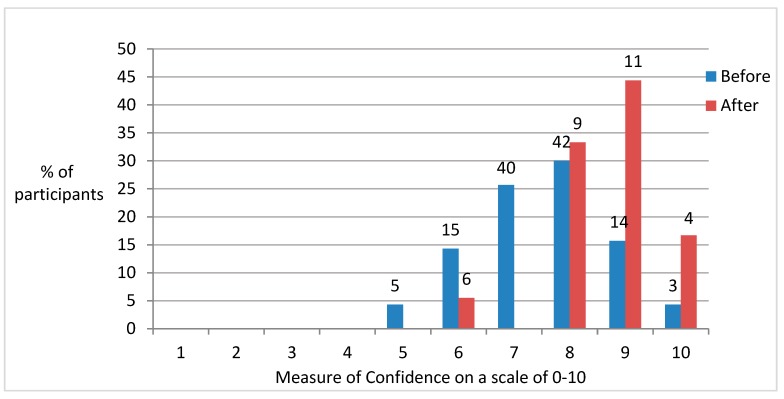
Changes in perceived confidence levels (where 0 = no confidence and 10 = total confidence) with respect to clinical skills in restorative dentistry before and after the programme.

Figure 1 and Figure 2 illustrate the level of confidence before participants started and on completion of the master’s programme, in relation to communicating with patients and ability to carry out dental procedures, respectively.

These figures demonstrated a general trend towards individuals becoming more confident after a prolonged period of study.

Table 3 illustrates that there was very little difference in confidence levels between the variants of age, gender and the number of years of clinical experience, but female participants were slightly more confident than their male counterparts (range 9–10; mean 9.25; SD = 0.500).

This is in contrast to the work of Mouatt et al., (1991) who reported a more significant difference in confidence between the genders with females being less confident than males [13] (pp. 76–79).

**Figure 3:**
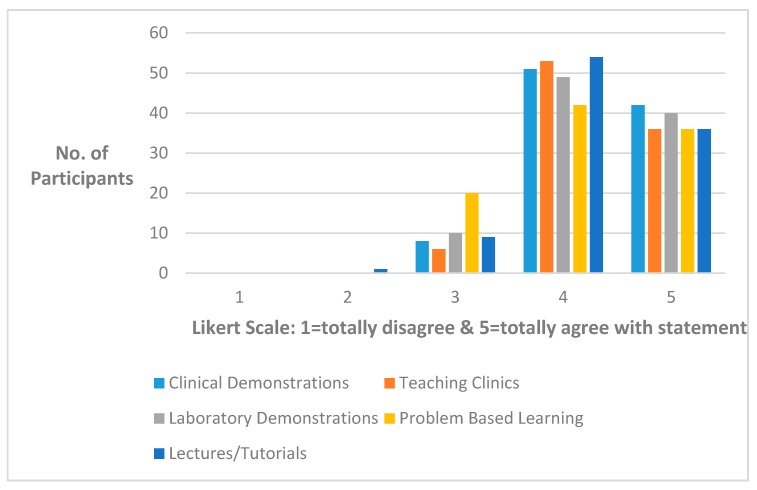
Preferred teaching method of the participants, following the master’s degree in restorative dentistry, in response to the statement ‘Which of the following teaching methods do you prefer?’.

As can be seen from Figure 3, the five main teaching methods were well thought of by the participants with most of them selecting 4 to 5 on a Likert scale [14] (pp. 1–55).

### 3.2. Qualitative Results

The participants’ decision to choose a particular university was based upon programme style, programme content, reasonable access to the facilities and the institute’s reputation. All these features were clearly expressed by the participants during the personal interviews.

All the participants reported an increase in their level of confidence in both communication and cognitive skills, following the programme. Very few participants reported setting themselves realistic, interim goals to progress through the programme. The thematic analysis of the qualitative data included major themes of confidence and self-efficacy; learning experience; motivation; and impact on practice.

A sample of the comments from individual participants included:

Confidence and self-efficacy:

**Respondent 2**: “*I do feel more confident because I can justify my treatment planning which is evidence based.*” This individual appreciated an increase in confidence and its ensuing implications of possessing greater scientific knowledge and using that to educate their patients.

**Respondent 4:** “*I think the fact that I have the knowledge background and possibly, hopefully, the skills to undertake what I need to do has made me more confident. Maybe at the same time I am probably more conservative in my treatment plans now*”. This participant tempered the increase in confidence obtained from greater knowledge with the realisation that sometimes intervention is not the best policy. This note of caution may be the result of a more prudent approach to clinical situations.

**Learning Experience**:

**Respondent 4:** “*I like learning with colleagues, the peer learning has a positive impact for me because you are always trying to do the best you can possibly do, especially if you have to consult with one of the others in a case for instance that you are struggling with, you want to make sure that your work is certainly up to a satisfactory standard before presenting them the case*”. The importance of peer learning, in the wider context of social learning, is displayed here.

**Respondent 9:** (Talking about which learning format is preferred) “*I think a mixture of all learning formats is good. It is nice to learn information in a lecture format and then it is nice to talk about things as well as a group. I think I prefer a combination of all teaching methods. We talked about cases during the hands-on sessions. There was a really good insight into how other people did things. Learning practical work, small groups worked better*”. A balanced approach is displayed here where several teaching methods were preferred to create a good learning environment for postgraduate dentistry.

**Motivation:**

**Participant 7:** “*I had just finished my MJDF (Membership of the Joint Dental Faculties, of the UK Royal Colleges) and was keen to build on that and do a certificate course and improve my skills. This option meant I could stay in practice and improve my skills*”. This participant illustrates the motivation to continue with their postgraduate education and that the programme allows that whilst being able to develop their skills in practice.

**Participant 8: “***My friend was my motivation. He applied and was the year above me at uni and he was saying that he was doing this restorative course and I thought oh I wouldn’t mind doing that, he said you may as well apply and I got in*”. This individual’s motivation seems to have come from a friend. Friends, colleagues and family can have a positive or negative impact on motivation.

**Impact on practice:**

**Respondent 5:** “*I wanted to be more confident in what I do and that is the one thing that this course has done for me, made me more confident in my clinical dentistry really*”. This participant achieved his/her objective to increase their confidence as a result of completing the programme. 

**Respondent 9:** “*So I have more confidence to take on bigger cases that I used to refer”.* The increase in confidence demonstrated here allowed the individual to treat more complex patient cases.

**Respondent 11:** “….*of course if you are confident about yourself then, the body language, the way you speak to them (patients). Yes I think I have taken on more work from treatment planning; more slightly expensive work*….” The individual here portrayed an enhanced level of self-efficacy with increased confidence and income.

## 4. Discussion

This study investigated the role that confidence had in respect to learning experience gained by participating in a long-term, part-time postgraduate dental education programme. Confidence is an important emotion, which is linked with motivation, self-efficacy, setting of goals and constructive feedback [4].

Most participants in this study highlighted increases in confidence, knowledge and clinical skills as the three main reasons for attending this postgraduate education programme.

Analysis of the quantitative data revealed that the participants reported an improvement in their perceived confidence, as measured on a linear scale. However, the anonymisation of the data did not allow for individual participants to be tracked throughout this cohort study, which was not considered to be a disadvantage since the results from the cohort [15] (pp. 235–388), rather than individuals, were the focus. The variables of gender and age did not appear to influence confidence. However, the number of years of post-qualification experience had a small negative impact on perceived confidence. Unexpectedly, the more experienced participants in this study were less confident than their less experienced counterparts. This might be due to (i) increased experience which generated higher levels of caution; (ii) greater seniority which led to feelings of inferiority relative to the younger colleagues who had a more contemporaneous training and (iii) increased experience which led to resistance to changes in clinical practice.

Analysis of the qualitative data showed that the learning experience met with the approval of the participants. In this context, the teaching methods (i.e. lectures/tutorials, clinical/laboratory/teaching sessions and problem-based learning/self-directed learning) proved to be appropriate and successful. The part-time nature of the programme was also seen by many of the participants (having completed different stages of the programme) to be advantageous for building confidence, citing: (i) opportunities to learn new skills and incorporate them into clinical practice; (ii) time to reflect on new skills and discuss them with colleagues; (iii) practical arrangements to allow participants to study and work at the same time; and (iv) opportunities to spread the cost of postgraduate education. The development of self-efficacy in the participants provided the motivational support for individuals to complete this challenging programme. Despite only one or two participants setting themselves goals to progress through the programme [16] (pp. 1017–1028), this cohort of GDP participants was able to successfully complete the programme.

In the training of health-care professionals, educators should be aware of this construct tendency and purposely promote student confidence in order to avoid a negative mindset. Construct tendency is the likelihood of individuals developing skills that will help with the learning process and allow them to fulfill their potential [17]. Teachers need to be able to assess levels of confidence and make improvements when it is low and, in comparison with objective measures of competence, address issues of misplaced over-confidence. Motivation, reinforcement and past experience are key components that promote self-confidence [18] (pp. 22–25).

Medical students tend to choose courses that suit their learning style which may facilitate their academic performance [19] (pp. 309–323). It has been suggested that ‘academic confidence has a role to play in predicting academic performance’ [20] (pp. 1–17).

It is clear that confidence varies amongst individuals in similar situations and that individual confidence is contextual in nature. The notion of confidence would therefore be subjective, personal and individual. What one individual reports as a feeling of confidence may be regarded by another as lacking in confidence. Individual variations will result in a range of emotions from lacking confidence to arrogance. 

Confidence is a perception of how comfortable the individual is to take on a task and should not be used synonymously with competence [21] (pp. 117–148). Competence is the ability to actually perform the task and is usually measured by another person, confidence is the individual’s belief of his/her ability. Assessments can be used as a tool to determine competence. Stewart et al (2000) also reported that an increase in skills (competence) resulted in an increase in confidence but not vice versa [21] (pp. 903–909).

The important contribution that the social aspect of learning has to offer should not be understated. Learning with colleagues who have similar ambitions and objectives, will allow participants to move forwards together, supporting each other and forming their own ‘community of practice’ [22] (pp. 98–100).

## 5. Conclusions

Following a five-year, part-time master’s programme in restorative dentistry, participants perceived improved knowledge and enhanced skills as a direct result of the learning experience. An overall increase in confidence and self-efficacy was observed. Perceived confidence, whilst difficult to quantify, was clearly increased.

It appears that the traditional approach to postgraduate dental education has value in improving the confidence of the participants and thereby their clinical knowledge and performance, to the ultimate benefit of patients. This cohort of GDPs recognised the effectiveness of traditional face-to-face teaching whilst developing individual self-regulated learning.

## Figures and Tables

**Table 1:** Information relating to the participants who completed the master’s programme.

Age	Range: 25–46 + Years Mean: 35
Gender	Male: 9 Female: 9
Number of Years Qualified	Range: 8–34 years; Mean: 14.2 years
Primary place of work	National Health Service Practice: Before the programme = 4 After the programme = 0
	Mixed NHS/Private Practice: Before the programme = 12 After the programme = 12
	Private Practice: Before the programme = 2 After the programme = 5
	Community Practice: Before the programme = 0 After the programme = 1
Level of Enjoyment on a scale of 0–10 (0 = no enjoyment; 10 = total enjoyment)	Range: 2–10; Mean: 6.9
Received a Satisfactory Learning Experience	Totally satisfactory = 6 Satisfactory = 9 Reasonably satisfactory = 2 Unsatisfactory = 1 Totally unsatisfactory = 0

**Table 2:** Illustration of the main reasons for attending the programme.

Reasons for Attendance	Percentage
To increase confidence	42%
To increase level of knowledge	41%
To increase level of skill	42%
To be up to date with cutting edge technology	29%

**Table 3:** The relationship between confidence (where 0 = no confidence and 10 = total confidence) and gender, age and number of years qualified.

Course Participant	Confidence Range	Mean	SD
**Gender**			
Male	7–10	9.00	1.069
Female	9–10	9.25	0.500
**Age**			
25–35 years	9–10	9.50	0.548
36–45 years	0	0	0
46+	7–10	8.67	1.033
**No. Years Qual.**			
2–10	9–10	9.20	0.548
11–20	9–10	9.20	0.447
21+	7–8	7.50	0.707
